# Chiroptical Strain Sensors from Electrospun Cadmium Sulfide Quantum-Dot Fibers

**DOI:** 10.1021/acsami.3c17623

**Published:** 2024-03-27

**Authors:** Hansadi Jayamaha, Thomas J. Ugras, Kirt A. Page, Tobias Hanrath, Richard D. Robinson, Larissa M. Shepherd

**Affiliations:** † Department of Human Centered Design, Cornell University, Ithaca, New York 14853, United States; ‡ School of Applied and Engineering Physics, Cornell University, Ithaca, New York 14853, United States; § Materials and Manufacturing Directorate, Air Force Research Laboratory, Wright-Patterson Air Force Base, Dayton, Ohio 45433, United States; ∥ UES, Inc., Beavercreek, Ohio 45432, United States; ⊥ Cornell High Energy Synchrotron Source, Cornell University, Ithaca, New York 14853, United States; # Robert F. Smith School of Chemical and Biomolecular Engineering, Cornell University, Ithaca, New York 14853, United States; ∇ Department of Materials Science and Engineering, Cornell University, Ithaca, New York 14853, United States

**Keywords:** electrospinning, quantum dots, cadmium sulfide, magic-sized clusters, strain sensors, chiral fibers

## Abstract

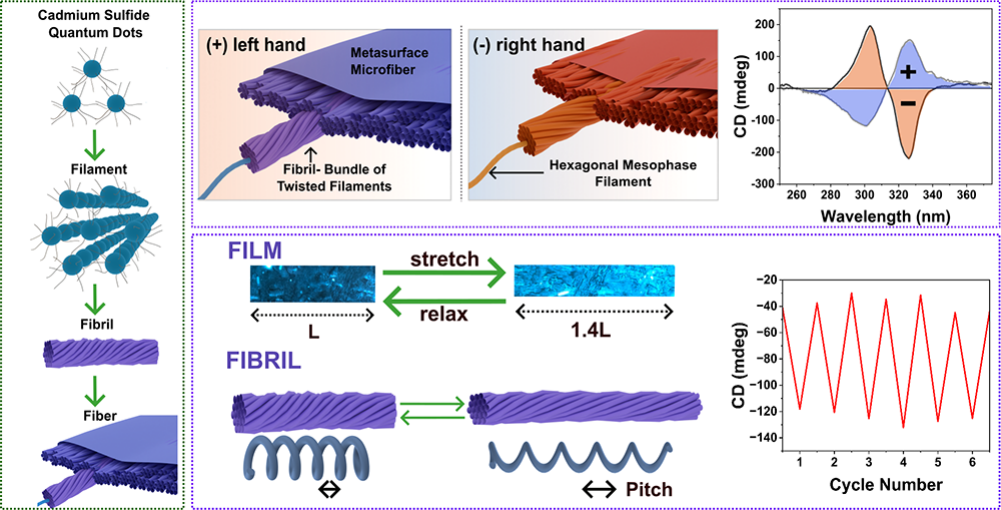

Controllable synthesis of homochiral nano/micromaterials has been a constant challenge for fabricating various stimuli-responsive chiral sensors. To provide an avenue to this goal, we report electrospinning as a simple and economical strategy to form continuous homochiral microfibers with strain-sensitive chiroptical properties. First, electrospun homochiral microfibers from self-assembled cadmium sulfide (CdS) quantum dot magic-sized clusters (MSCs) are produced. Highly sensitive and reversible strain sensors are then fabricated by embedding these chiroptically active fibers into elastomeric films. The chiroptical response on stretching is indicated quantitatively as reversible changes in magnitude, spectral position (wavelength), and sign in circular dichroism (CD) and linear dichroism (LD) signals and qualitatively as a prominent change in the birefringence features under cross-polarizers. The observed periodic twisted helical fibrils at the surface of fibers provide insights into the origin of the fibers’ chirality. The measurable shifts in CD and LD are caused by elastic deformations of these helical fibrillar structures of the fiber. To elucidate the origin of these chiroptical properties, we used field emission-electron microscopy (FE-SEM), atomic force microscopy (AFM), synchrotron X-ray analysis, polarized optical microscopy, as well as measurements to isolate the true CD, and contributions from photoelastic modulators (PEM) and LD. Our findings thus offer a promising strategy to fabricate chiroptical strain-sensing devices with multiple measurables/observables using electric-field-assisted spinning of homochiral nano/microfibers.

## 1 Introduction

Self-assembly and chirality are prevalent natural phenomena found in a variety of structures, including proteins, DNA, and RNA, as well as in nanoscale photonic structures observed in butterfly wings and macroscopic structures seen in conches.^1^ Many studies are being carried out to mimic self-assembly and create chiral nanomaterials through novel synthesis and manipulation techniques.^1−4^ The chiroptical properties of these intrinsically chiral nanomaterials are affected by internal and external stimuli such as phase change,^5^ magneto-optical effect,^6^ and mechanical deformation,^7,8^ demonstrating their promising potential as sensing devices for optical,^5^ biological,^7,8^ and catalytic systems.^9^

One particular area of interest is optical strain sensors, which are dynamic systems with photoelastic properties for analysis of spatial stress distribution across materials and systems including biomedical devices and architectural structures.^7,8,10^ In this application, material choice is challenging as an ideal material will require minimal stress-shielding effects and sufficiently large photoelastic responses for accurate localized strain measurements^11^ and/or measurable shifts in the phase, magnitude, and/or spectral position of circular dichroism (CD) signals.^8^ Presently, these sensors rely on extremely sensitive measurements of photon time-of-flight as reflected by fiber Bragg gratings (FBGs) in glass or plastic fibers,^12^ or intensity modulation in elastomeric ones.^13^ The use of alternative optical signals, such as CD, would allow for the encoding of more information in the same waveguides.

While various unique chiral nanomaterial-based superstructures have been prepared, few studies report continuous chiral fibers as promising candidates for chiroptical devices.^21^ Of the chiral fibers reported, they are composed of polymer and chiral nanoparticles,^14^ helical polymers,^15−18^ and organic/inorganic nanocrystals.^19−21^ One challenge of fibers, when compared with their film counterparts, is achieving strong “true” CD signals. Commonly, the CD measurements obtained from a CD spectrometer contain contributions from the ordered orientation of transition dipoles (known as Linear Dichroism—Linear Birefringence, LDLB, contributions) and/or residual static birefringence arising from imperfections in photoelastic modulators (PEM).^22,23^ These are collectively referred to as linear anisotropy-induced CD or “apparent” CD. Although only considered in a few studies, these contribute to a major portion of the CD measurements for fibers.^15,23^ Therefore, this study considers both the apparent and the true CD signals for fibers.

Electrospinning, a method for fiber production, can be used to finetune the structure and orientation at the molecular level to achieve the required properties and performance at the macroscopic level.^24,25^ This technique is versatile, cost-effective, and routinely used to produce 1D and 2D nanomaterials with a high surface-to-volume ratio.^26^ We have previously produced unaligned electrospun fibers from oleic acid-capped cadmium sulfide (CdS) to achieve hierarchical assembly across multiple length scales;^27^ however, uniaxially aligned fibers are necessary for specific applications including optical devices and biomedical implant devices.^28,29^ To achieve aligned fibers using electrospinning, a rotating collector, copper wire drum, scanning tips, or conductive plates may be used.^30^ In this study, we used a rotating drum for the alignment of fibers to enhance their chiroptical properties.

This study focuses on the synthesis and analysis of CdS MSC fibers through electric-field-assisted nano/microlevel assembly. Macroscopic level alignment is achieved by adjusting electrospinning parameters. Unlike the mechano-responsive chiral materials reported in the literature,^8,31−35^ this has enabled predominately homochiral fibers, which are then embedded in elastomeric films to demonstrate reversible photoelastic properties and quantifiable shifts in the chiroptical activity over multiple mechanical extension/relaxation cycles. We also isolate the true CD from the measured CD using a method derived in literature,^36^ confirming the chiral assembly of the MSC mesophase within the electrospun fibers and the utility of these electrospun homochiral fibers as chiroptical strain-sensing devices.

## 2 Results and Discussion

### 2.1 Hierarchical Assembly of MSCs Using Electrospinning

To synthesize ultrapure CdS MSCs, we used a previously reported, high-concentration synthesis method.^37,38^ The resulting MSCs are composed of CdS quantum dots, each with a diameter *D*_QD_ ∼ 1.5 nm. They have core Cd atoms exhibiting a tetrahedral coordination with S but with an overall low-symmetry molecular structure owing to their ultrasmall size. These MSCs assemble into a mesophase with nanometer spacing between units both radially and axially.^38^ Controlled evaporation of the solvent has been shown to lead to the formation of highly ordered films with periodic structures.^37^ Specifically, these mesophase filaments align in a shear flow field, resulting in the formation of periodic bands/textures.^37^ In our case, the many-fold faster evaporation and shear alignment from the nozzle during fiber formation results in assembly along the continuous fiber axis.

In this study, we electrospin the CdS/chloroform suspensions via two approaches: (i) a traditional electrospinning setup with a stationary collector as described in our previous work,^27^ and (ii) a modified electrospinning system using a rotary drum collector at rotary speeds of 2500 and 3200 revolutions per minute (rpm); Figures S1 and S2a,b provides rheological data and the electrospinning setups, respectively. For additional details, we also refer the reader to a recent study that mentions the effect of fiber diameter, porosity, and film thickness on chiroptical properties, such as LDLB and true CD, that is not covered within the scope of our study.^23^

**Figure 1:**
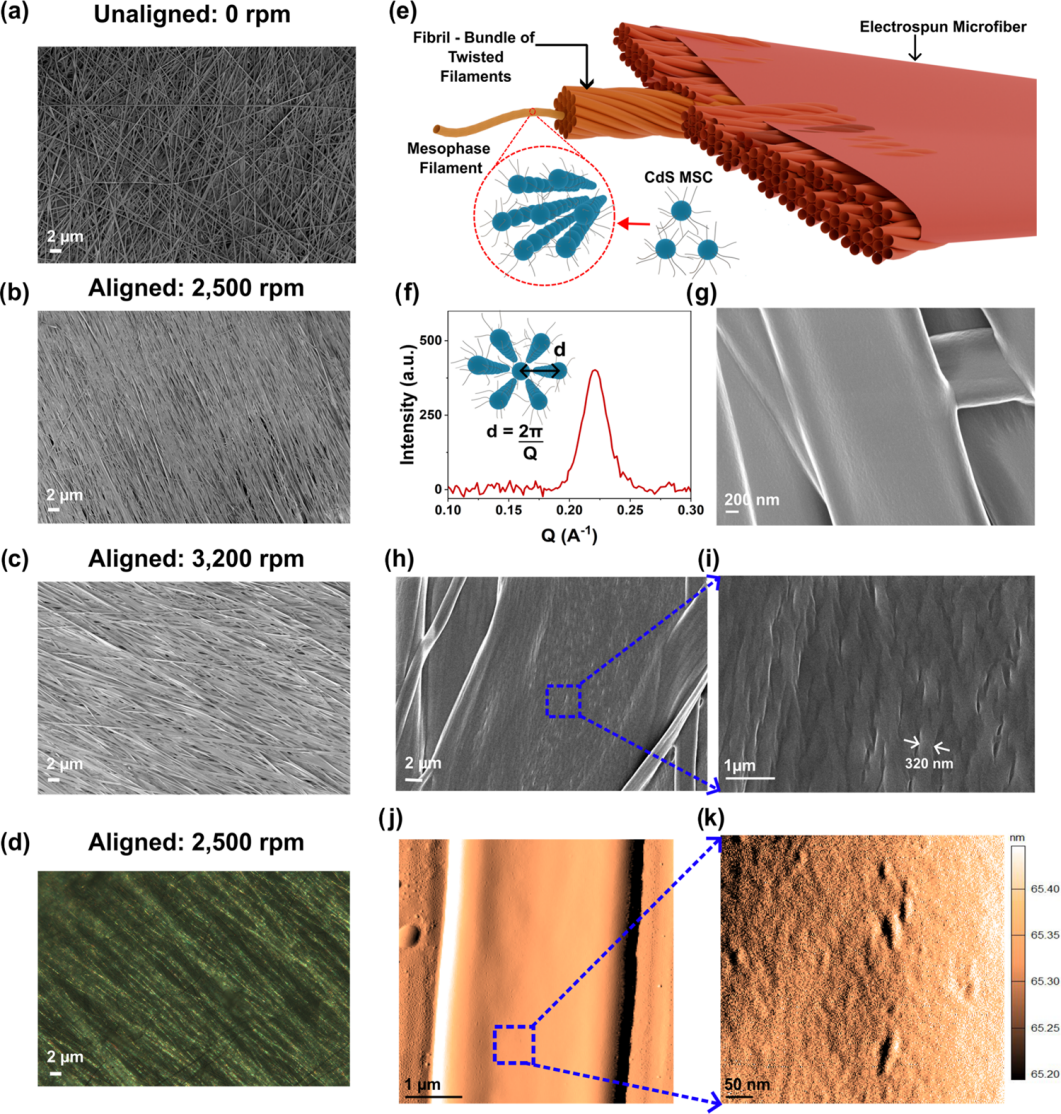
FE-SEM image of (a) randomly oriented fibers from the stationary collector, (b, c) well-oriented fibers at rotatory speed −2500 and 3200 rpm, and (d) polarized optical microscopy image of fibers at rotatory speed 2500 rpm, (e) Schematic of the proposed hierarchical structure of the ribbon shaped microfiber, (f) SAXS of the fiber mat, inset–hexagonal structure of the mesophase and the inter mesophase filament spacing, SEM images of (g) fibers with smooth surfaces from collector rotating at 2500 rpm, (h, i) fibers from collector rotating at 2500 rpm with larger twisted fibrils at the surface, (j, k) AFM amplitude measurement of fibers with the smooth surface having striations or much smaller nanofibrils along the fiber axis (scale indicates the variation in height).

We aligned CdS MSC microfibers parallel to each other on a macroscopic level (collection area, *A*_macromat_ ∼ 30 × 3.4 cm^2^), by using the rotary drum, thus incorporating an additional hierarchical level of assembly (Figure 1a–c). The uniaxial shearing between the needle and the collector (induced by electrostatic forces and uniaxial elongational flow)^39^ along with the radial solvent evaporation front cause self-assembly of the liquid crystal (LC) like MSC mesophase along the fiber axis. The LC nature of CdS MSCs and the orientation of the mesophase along the fiber axis are evident from the birefringence features observed in fiber mats under the polarized optical microscope (Figure 1d). The mesophase orientation along the fiber axis is further confirmed with the Herman’s orientation factor (HOF). The HOF ranges from 0 to 1, with 0 referring to no alignment, and 1, perfect alignment. For fiber mats spun on a rotating drum collector at 2500 rpm, based on SAXS experiments, the HOF is ∼0.79, which suggests that the mesophase is well oriented along the fiber axis (Figure S3 and eqs S1 and S2).

The evaporation processes in electrospinning, like in films, give rise to higher-ordered self-assembled structures.^37,38^ Based on our findings, we propose a hierarchical assembly of MSCs within these electrospun fibers (Figure 1e). We also refer the reader to studies on wool fibers for information and generally used terminology on the hierarchical composition of fibers.^40^ We used SAXS experiments to calculate the d-spacing between MSCs to be 2.84 nm and the lattice constant for the (100) plane to be 3.25 nm (*q* ∼ 0.221 Å^–1^). The mesophase grain size is ∼15 nm (11% of the peak width is from instrument broadening; refer Figure 1f; see eqs S3 and S4). The lattice constant corresponds similarly to the value reported for the hexagonal mesophase assembly observed in film form.^38^ The similar lattice constant observed in fibers and films supports our hypothesis that the self-assembled mesophase structure is not disrupted by electrohydrodynamic forces during electrospinning. Of note, the mesophase grain size is extremely small (even smaller than in films^38^). This is due to the electric-field-assisted assembly of the microfibers in contrast to films.

The mesophase filaments bundle together into larger fibrils.^41^ These fibrils assemble into larger twisted structures while showing long-range alignment along the electrospun fiber axis. The surfaces of most fibers from field emission scanning electron microscopy (FE-SEM) images are smooth or slightly rough with nanoscale fibrillar structures (Figure 1g); however, we observe twisted fibrils in relatively larger fibers (>5 μm, Figure 1h,i). The twisted fibril-like morphology forms during self-assembly to yield higher stability by the relaxation of additional stresses introduced by the faster solvent evaporation rate of electrospinning. The reason for the occasional appearance of significantly larger twisted fibrils on some fibers can be explained as the result of inconsistencies in fiber elongation due to the variation in drum speed (up to ±5% rpm from the set speed) that occasionally results in larger fibers.

The morphology of the fibers in FE-SEM and atomic field microscopy (AFM) are ribbons with the aligned fibers spun at 2500 rpm having a thickness of *T* ∼ 60.7 ± 13.7 nm with slightly raised edges. This morphology is a result of rapid solidification of the fiber sheath, which leads to the collapse of the spherical fiber structure (Figures 1j and S4).^27^ A stationary collector results in a random distribution of fibers on the collector surface with the diameter of the electrospun microfiber, *D*_microfiber_ = 2.30 ± 0.33 μm. Our use of a rotary drum at 2500 and 3,200 rpm has resulted in slightly larger fibers (*D*_microfiber_ = 2.82 ± 0.48 and 2.92 ± 0.58 μm, respectively). Increasing the drum′s collection speed to 3200 rpm does not increase the uniaxial alignment nor significantly reduce the fibers’ diameters. While these observations contrast with what is generally observed in electrospinning conventional polymers, we believe at short collection distances (∼7 cm) and increased airflow from the rotating drum, the evaporation rate of the chloroform is increased, resulting in more rapid fiber solidification and less thinning upon collection.

Our AFM analysis of the fibers further reveals a metasurface with striation features along the fiber axis with a spacing of *D*_striation_ ∼ 24 ± 3 nm, confirming the presence and alignment of fibrils composing the microfiber (Figure 1j,k). These fibrils are smaller (*D*_fibril_ ∼ 15 ± 2 nm) than what is observed in FE-SEM images of larger fibers (*D*_fibril_ ∼ 336 ± 40 nm). The size of the smaller fibrils (*D*_fibril_ ∼ 15 ± 2 nm) corresponds similarly to the mesophase grain size calculated from the X-ray scattering data. Regardless of the dimensions of the twisted fibrils, we measure significant chiroptical properties from MSCs, where chiroptical activity refers to the selective absorption or dispersion of circularly polarized light by dissymmetric materials that may or may not possess chirality.^42^

### 2.2 Chiroptical Properties of MSC Fibers and Effect of Electrospinning

**Figure 2:**
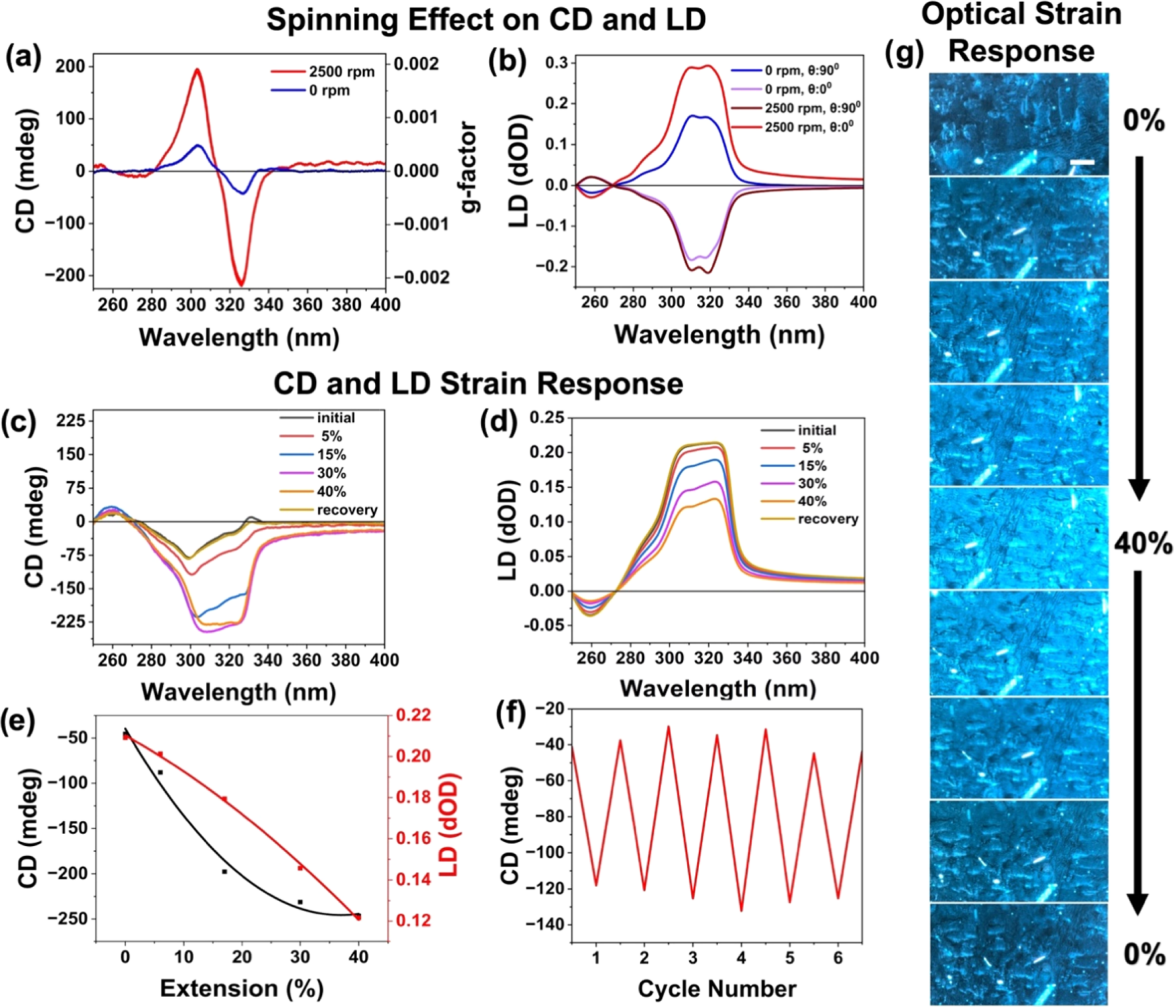
Chiroptical properties of electrospun fiber mats produced using a stationary collector and rotary collector at 2500 rpm (a) Average CD and *g*-factor, (b) LD (fibers oriented vertically and horizontally), (c, d) CD and LD for fibers embedded in PDMS film (length 2 cm) at different elongation levels up to 40 ± 2% and relaxed to original length, (e) magnitude of CD and LD at 310 nm for fibers embedded in PDMS film (length 2 cm) plotted against extension% given as a scatter plot and 2nd order polynomial fitting for the data points, (f) cyclic response of CD measured at 300 nm over 6 cycles of stretching and relaxing and (g) Polarized optical microscope images of MSC fibers embedded in PDMS film (i.e., MSC/PDMS films) while being stretched up to 40% and relaxed to its original length (scale bar is 50 μm).

The chiral properties of these fibers are studied using CD spectra. Our fibers share a bisignate profile centered around the excitonic peak at λ ∼ 314 nm, indicating that the assemblies adopt a chiral arrangement and form an exciton-coupled system (Figure 2a). These excitonic peaks have been previously shown for β-CdS and α -CdS isomers at λ ∼ 313 and λ ∼ 324 nm, respectively (Figure S5).^43^

To determine the orientation of the mesophase in the fibers, we used measurements of linear dichroism (LD). In the case of absorptive dichroic polarizers, the most used linear polarizers, the optical polarization results from the linear alignment of electronic transition dipoles.^44^ LD is only present around the exciton absorption wavelength (Figure 2b); therefore, the alignment of the electronic transition dipoles of the MSCs is the source of the LD, indicating again that the mesophases are aligning along the fiber axis.

The higher degree of fiber orientation from the rotary collector compared to the stationary collector increases the true CD by more than 3-fold (Figure 2a). Chiral assembly within fibers is therefore affected by the orientation, increasing shear, and rapid evaporation due to winding during electrospinning. We removed the effect of concentration on the CD signal by calculating the *g*-factor, which is the true CD divided by the absorption. Note that the linearly aligned MSC fibers (2500 rpm) possess *g*-factors at *g* ∼ 0.002, which is 10-fold higher than unaligned fibers and close to values reported for spin-coated films at 0.0037.^37^ Increasing the rpm up to 3200 rpm yielded far less alignment and lower *g*-factors due to collector drum instabilities (Figure S6). Therefore, for fabricating the optical strain sensors, due to the higher CD and *g*-factors, we used the well-oriented fibers obtained at 2500 rpm.

MSC solutions result in either a positive or negative bisignate profile (Figure S5d), indicating the presence of the two enantiomorphs (or handedness) reported in the literature.^43^ In our electrospinning deposition, however, we measured strongly homochiral samples across the entire fiber mat. By measuring the CD at different positions across the fiber web, we measured the same handedness (either positive or negative) with a fractional probability of ∼0.8 (Figure S7). Therefore, we believe electrospinning results in electric-field-assisted unidirectional twisting of the mesophase fibrils, although further studies are needed to confirm the exact effect of the electric field on the MSC rearrangement and assembly as fibers. Interestingly, we were not able to control the exact enantiomorph obtained from electrospinning parameters, collector rotation direction, or speed. Accordingly, we believe that the exact handedness of the fiber mat depends on the initial synthesis steps of the MSCs and/or ambient conditions such as relative humidity and temperature during electrospinning and storage. Regardless, the degree of homogeneity in our electrospun mats is much greater than that observed in films, which generally have random distributions of handedness.

### 2.3 Strain-Induced Changes in Chiroptical Properties

To impart mechanical resilience into these oriented fiber mats, we imbibed them in poly(dimethylsiloxane) (PDMS). The resultant fiber composite strips (MSC/PDMS films) show strain-sensitive CD response on stretching along the fiber axis (Figure 2c) and no significant CD change on stretching in a direction perpendicular to the fiber axis (Figure S8). This observation further validates our hypothesis that the twisted fibrillar structure or helical axis is arranged along the fiber axis. We attribute the ability to affect the chirality and photoelastic properties, to changes in the helical pitch upon stretching along the fiber axis, although contributions from LDLB cannot be overlooked. It is worth noting that the LD reduces in both stretching directions likely due to local misalignments of the electron dipole moments. In principle, the LDLB artifacts observed during stretching can be also explained by these local dipole reorientations along the birefringent axis (i.e., the fiber axis) during stretching.

We observe the gradual changes in magnitude, sign, and peak position of the CD signal and reduction in the LD signal on stretching the chiroptical film along the fiber axis up to 40% (Figure 2c,d). We fit the change in magnitude of the CD and LD under strain (ε), 0% < ε < 40%, at λ ∼ 310 nm using a second-order polynomial function (Figure 2e). After the initial four cycles of strain softening of the nanoparticle-elastomer film (see Mullins effect^45,46^), the CD response shows almost complete recovery over an additional 6 cycles (Figures 2f and S9). The strain response of the film is also observed under the polarized optical microscope as a clear shift in brightness as the film is stretched (Figure 2g and SI Video). Based on these findings, the detection range of the sensor can be defined as 4.5–40%, with a nonlinear response within this range (Figure 2e). Below ε ∼ 4.5%, the CD response appears to be ambiguous and can be attributed to thickness variations rather than a mechano-responsive chiroptical activity (Figure S9d). The spring-like nature of the MSC fibrils results in a nonlinear response and the measured CD and the sensitivity plateaus at the detection upper bound of 40%. Therefore, the sensitivity of the sensor, which is given by the relative rate of change of CD with the extension,^47^ also suggests a nonlinear response (Figure S9e and eq S5).

**Figure 3:**
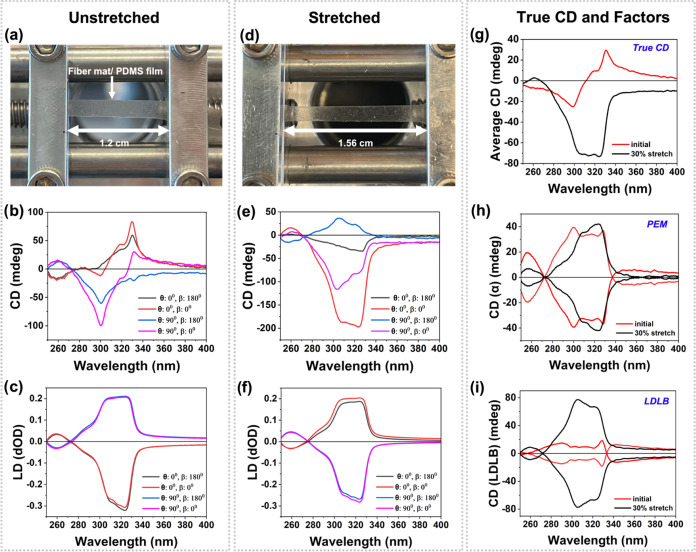
Fibers electrospun using the rotating collector at 2500 rpm, imbibed in PDMS elastomer film and their 4-scan CD and LD for (a–c) initial and (d–f) 30% stretched samples, (g) true CD (or “average CD”) and the contributions from (h) PEM and (i) LDLB.

To further corroborate the reasoning behind this strain-sensitive response and the true chirality of the fibers, we assumed our samples to be continuous and homogeneous and isolated the true CD from the measured CD for samples before and after stretching by using the 4-scan method reported by Yao et al.^36^ (Figure 3). The true CD is calculated (eq 1) by taking the average of the measured CD of the sample at 4 different orientations (Figure S10).
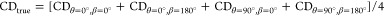
1Changes in θ refer to whether fibers are aligned horizontally (θ = 0°) or vertically (θ = 90°), while β refers to whether fibers are facing toward (β = 0°) or away (β = 180°) from the UV source of the CD spectrometer. The complete set of equations are given in eqs S6–S9.

The antisymmetric CD_α_ and CD_LDLB_ suggest that the 4-scan method was accurately applied for our system (Figure 3h,i). These findings indicate the applicability of the method for fiber mats with porosity and for fibers imbibed in achiral polymer films (even though the Mueller matrix assumes the system is homogeneous and nondepolarizing).

The strong LDLB contributions isolated from the 4-scan data are noteworthy as it confirms LDLB affects the CD signal on stretching (Figure 3i). According to the literature, changes in the CD signal on rotating the sample are also commonly attributed to LDLB effects.^18^ That is, when the sample is flipped, the sign between the LD and LB principal axes flips, inverting the sign of the term in the expression for measured CD.^48−50^ We do not observe the CD signal inverting when the sample is rotated by 180° around its vertical axis (Figure 3b); however, the bisignate shape disappears, further validating the LDLB contribution, while the true CD confirms the chirality of the fibers (Figure 3g).

**Figure 4:**
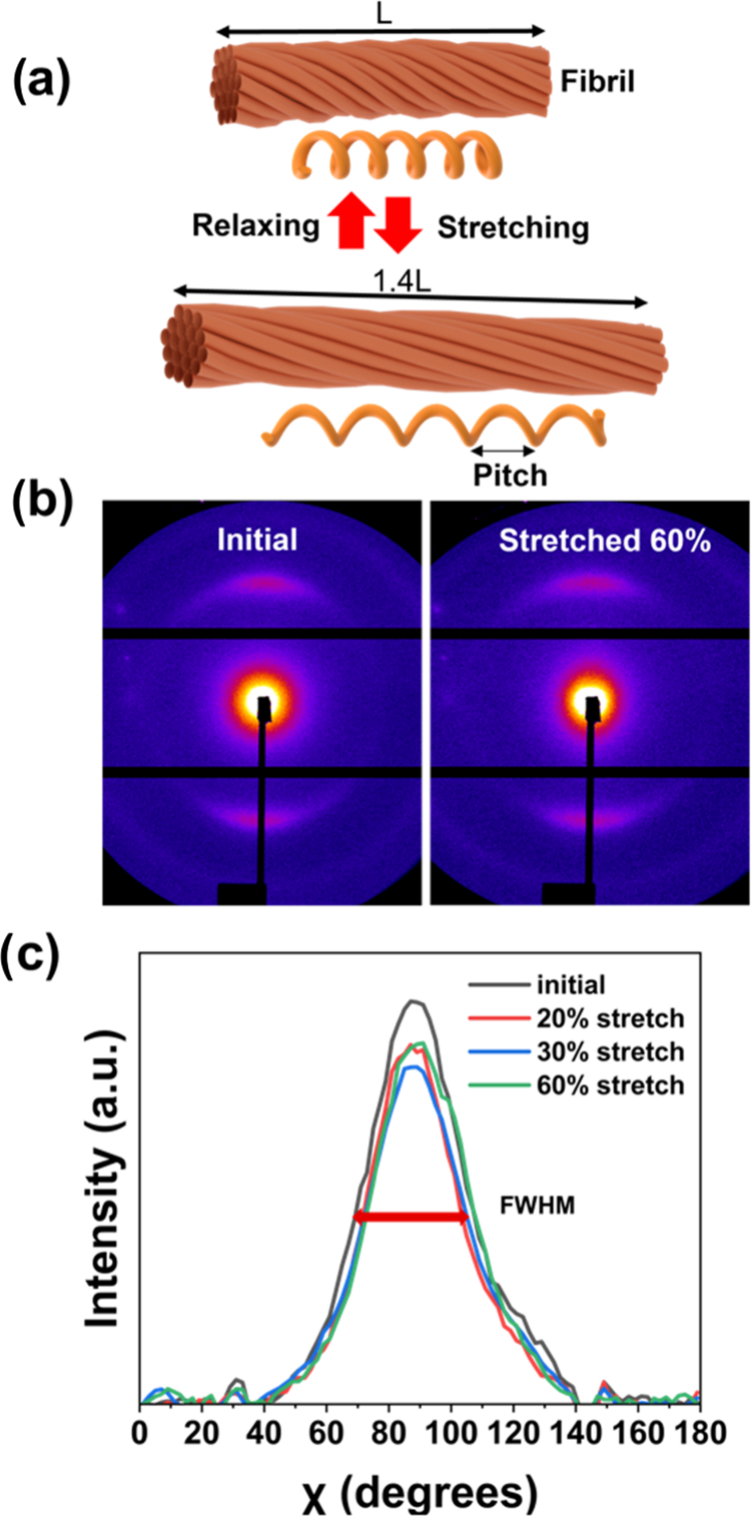
(a) Illustration of a single fibril being stretched and the increasing pitch. (b) Scattering pattern for MSC/PDMS film (where the fibers are oriented parallel to the horizontal axis) and stretched at 60% and (c) azimuthal scan for MSC/PDMS film at different strains (up to 60%).

CD and photoelastic response, therefore, can be linked to the true chirality of the fibers as evidenced from the bisignate shape observed in the initial “true CD” (Figure 3g). LDLB artifacts obfuscate the CD signal upon stretching, resulting in an incomplete flip of the CD signal. Due to the reversible shifting of the CD peak from λ ∼ 331 to 324 nm (Figures 2c, 3g, and S9a) and the enhanced birefringence features, we confirm there is an increase in the helical pitch (or helical angle) of the fibril structure (Figure 4a), although there is no evidence to suggest a change from one enantiomorph to the other.^44^ To further support this explanation, we provide the HOF and the radial/axial *d*-spacing of the mesophase filaments that do not change upon stretching. These demonstrate that the average mesophase orientation and filament spacing are not affected by stretching (Figures 4b,c and S3 and Table S1). We have therefore confirmed that the only possible structural change is the increase in the helical pitch of the fibrils.

## 3 Conclusions

Electrospinning-induced self-assembly can be used to create homochiral microfibers, enabling promising integration of these fibers as chiroptical devices. The organic–inorganic MSCs allow carrier-free spinning of highly aligned fibers using a rotary collector. The additional forces at work during electrospinning allow for unidirectional twisting of the mesophase, causing a consistent handedness at different locations across the fiber web. This uniformity in handedness contrasts with films, where from the same deposition, we see a random distribution of handedness. In this sense, electrospinning-induced self-assembly is superior for producing monolithic CD features with comparable g-factors to spin-coated films.^21^

We then embedded the chiral fibers into an achiral polymer matrix to form stretchable chiroptically active films with a high strain sensitivity. Stretching and relaxing of the MSC/PDMS films show strain-sensitive and reversible flipping and shifting of the excitonic CD signal and a decrease in the LD signal. The blue-shifting of the CD peak is indicative of alterations in the helical pitch of the fibrillar structures within the fiber. Additionally, the liquid crystalline nature of the MSC also brings about noticeable shifts in birefringence, as the MSC/PDMS films are stretched under cross-polarizers. The combination of these effects offers a new opportunity in the design and fabrication of strain sensors with multiple strain-induced responses for both quantitative and qualitative deformation mapping of dynamic systems.

## 4 Experimental Details

### 4.1 Materials

Chloroform was purchased from VWR and TCE. SILASTIC MS-4007 Moldable Silicone was supplied by Dow chemical company Pvt. Ltd. Materials and methods used to synthesize Cadmium Oleate and CdS MSCs are described in previous work.^37,38^ All reagents were used as received without further purification.

### 4.2 Electrospinning

CdS MSCs (3.3 wt %) were added to chloroform and mechanically agitated for 24 h (using a laboratory shaker) to form a gel-like suspension Figure S11a,b. The well-dispersed MSC suspension was then electrospun as described here. A 1.5 in. long, 20-gauge disposable nonbeveled needle (Nordson EFD LLC) was fitted to a 5 mL glass syringe (Chemglass). The voltage was maintained at 15 kV (γ High Voltage Research Inc.) A flow rate of 2.5 mL/h was then applied using the PH Ultra syringe pump (Harvard Apparatus). The suspension was directly electrospun onto a copper (Cu) sheet wrapped around a grounded drum collector (MSK-DC-3000, MTI Corporation) or attached to a stationary collector plate (Figure S11c). The gap between the needle tip and the collector surface was 7 cm. For CD Spectroscopy and SAXS measurements, slits were made on the Cu sheet to collect the fiber webs (Figure S11d). The rotation speed of the drum collector was maintained at 2500 and 3200 rpm (with ±5% variation) to study the effect of alignment of fibers on chiroptical properties.

### 4.3 MSC/PDMS Strain Sensor Preparation

First, the fiber mat is electrospun onto a Cu plate wrapped around the rotary drum collector. Then SILASTIC MS-4007 Moldable Silicone (PDMS) is doctor-blade coated on the Cu plate with fibers and allowed to cure at 40 °C overnight. The choice of the polymer was based on the achiralily and photoreflectivity effects of PDMS (Figure S12). The MSC/PDMS film is then peeled off the Cu plate. Strips of 0.5 cm × 2 cm were cut to be analyzed using the CD spectrometer for strain response studies.

### 4.4 Circular Dichroism (CD) Spectrometer

CD and LD measurements were conducted using a JASCO J1500 CD spectrometer. The beam in this instrument is elliptical, and the exact beam size depends on the bandwidth. Therefore, to remove the beam inhomogeneity, a 1 mm diameter circular aperture is installed. The Cu sheet had a rectangular slit with the fibers attached to the aperture and fitted onto the instrument stage. To study the changes in CD and LD on stretching the elastomeric films with the fibers, a simple tensile stage (Figure S13) was fabricated in-house. This allowed clamping of the films and gradually symmetrical stretching of the samples. Measurements were then taken of the stretched films. A circular aperture with a diameter of 2.5 mm was used at this stage to remove the inhomogeneity of the beam. For each measurement, CD, LD, and absorbance were recorded.

### 4.5 Small Angle X-ray Scattering (SAXS)

These measurements were carried out at the Functional Materials Beamline (FMB) of the Materials Solutions Network at the Cornell High Energy Synchrotron Source (MSN-C). An X-ray beam energy of 9.7 keV (λ *=* 1.28 Å) was selected using the 111 reflection of a single-bounce, HPHT diamond monochromator.^51^ Harmonic rejection and vertical focusing are provided by a 1 m long, bendable, rhodium-coated monochromatic mirror located approximately 7 m upstream of the experimental hutch at an incident angle of 4 milliradians. Experiments were carried out in “bulk-beam” mode, and the monochromatic mirror was used to focus the beam into a spot approximately 0.08 × 0.5 mm^2^ at the sample position, with a total flux of approximately 10^12^ photons/second at 100 mA beam current. Scattering images were collected on a Pilatus 300 K detector (Dectris, Baden, Switzerland) with a sample-to-detector distance of ca. 72 cm and equipped with a diode beam-stop. Detector images were azimuthally integrated to produce intensity versus scattering vector, *Q*(Å^*–*1^), plots that were then corrected for both incidence beam flux and sample absorption. All calculations and baseline correction of data were carried out in Origin(Pro), Version (2019) OriginLab Corporation, Northampton, MA.

### 4.6 Rotational Shear Rheometer

The flow properties of the CdS MSC dispersed solution were determined using a DHR3 Rheometer with a 20 mm diameter, 2° cone plate. A flow rate sweep was recorded at a constant temperature of 21 *°*C for solutions from fresh and aged MSC samples.

### 4.7 Field Emission Scanning Electron Microscope (FE-SEM)

The microstructure of CdS fibers was studied using the Zeiss Gemini 500 FE-SEM. The fibers were sputtered with gold palladium for 40 s before imaging at a 1 kV accelerating voltage. Diameters of fibers and fibrils were measured manually by using ImageJ. A 30-fiber and 10-fibril count is used to measure the average diameter and relative diameter distribution. The thickness was measured considering 10 fibers, and an average was reported.

### 4.8 Atomic Force Microscope (AFM)

The nanosurface structure of the electrospun fibers was gathered using amplitude images in AC mode using an Asylum Research MFP-3D AFM. AFM measurements were carried out on samples electrospun onto Cu sheets attached to glass slides. The diameter and interfibril distance of fibrils were measured manually using ImageJ. Each measurement was taken at 10 spots, and the average and standard deviation were recorded.

### 4.9 Cross-Polarized Optical Microscope

Optical images were taken by using an Olympus BX51 cross-polarized microscope. Linkam tensile stage (controller model T 96-S) was fitted with the microscope, and videos/photos were recorded/captured as the film was stretched. The polarization filter is set at an angle where the extinction features (dark image) of the film appear and then gradually stretched up to 40% at a constant velocity of 50 μm per second. Pristine PDMS film of the same thickness is also stretched under the same conditions to invalidate any effects of the thickness variation on the brightness of the image (Figure S14).

## Supporting Information Available

Figure 1: Viscosity vs shear rate and spinnability, Figure 2: Traditional electrospinning setup Vs. setup with rotary drum collector, Figure 3: Herman’s orientation factor, Figure 4: Ribbon shape of the electrospun fibers (SEM), Figure 5: Isomers and Enantiomorphs of MSCs, Figure 6: Chiroptical properties of fibers wound at 3200 rpm, Figure 7: Homochirality observed from a single batch of MSCs, Figure 8: Chiroptical response on stretching the MSC/PDMS film across the fiber axis, Figure 9: Cyclic CD and LD response on stretching along the fiber axis, Figure 10: 4-scan method to isolate true CD, Figure 11: Electrospinning collector setup for spinning fibers, Figure 12: Chiroptical properties of PDMS films, Figure 13: In-house made tensile stage for studying chiroptical properties while stretching MSC/PDMS films, Figure 14: Changes in birefringence features on stretching PDMS and PDMS/MSC films and Calculations of Herman’s orientation factor, instrumental peak broadening in SAXS, Scherrer Calculation and detector sensitivity (PDF)
The strain response of the film is also observed under the polarized optical microscope as a clear shift in brightness as the film is stretched (SI Video) (MOV)

## Supplementary Material

am3c17623_si_001.pdf

am3c17623_si_002.mov

## Abbreviations

MSC: magic-sized cluster
CD: circular dichroism
LD: linear dichroism
LDLB: linear dichroism liner birefringence
FE-SEM: field emission scanning electron microscopy
AFM: atomic force microscopy
PEM: photoelastic modulators
FBG: fiber Bragg gratings
LC: liquid crystal
HOF: Herman’s orientation factor
PDMS: poly dimethylsiloxane
