# Supplementary material for: Chiroptical Strain Sensors from Electrospun Cadmium Sulfide Quantum-Dot Fibers
Source: ACS Appl Mater Interfaces. 2024 Mar 27;16(14):17757–65. doi: 10.1021/acsami.3c17623 (PMC11009915; doi:10.1021/acsami.3c17623)
Supplement: Supplementary file 1 — am3c17623_si_001.pdf [file am3c17623_si_001.pdf]

Chiroptical Strain Sensors from Electrospun Cadmium Sulfide Quantum-dot Fibers

Hansadi Jayamaha[1], Thomas J. Ugras[2], Kirt A. Page[3,4,5], Tobias Hanrath[6], Richard D. Robinson[7] and Larissa M. Shepherd[1]*

[1]Department of Human Centered Design, Cornell University, Ithaca, New York 14853, United States

[2]School of Applied and Engineering Physics, Cornell University, Ithaca, New York 14853, United States

[3] Materials and Manufacturing Directorate, Air Force Research Laboratory, Wright-Patterson Air Force Base, Ohio 45433, United States

[4]UES, Inc., Beavercreek, Ohio 45432, United States

[5]Cornell High Energy Synchrotron Source, Cornell University, Ithaca, New York 14853, United States

[6]Robert F. Smith School of Chemical and Biomolecular Engineering, Cornell University, Ithaca, New York 14853, United States

[7]Department of Materials Science and Engineering, Cornell University, Ithaca, New York 14853, United States

Supplementary Information

Table of Contents

Figures and Tables 2
Viscosity and spinnability 2
Traditional electrospinning setup Vs. setup with rotary drum collector 3
Herman's orientation factor and orientation angle 4
Ribbon shape of the electrospun fibers (SEM) 5
Isomers and Enantiomorphs of MSCs 6
Chiroptical properties of fibers wound at 3200 rpm 7
Homochirality observed from a single batch of MSCs 8
Chiroptical response on stretching the MSC/PDMS film across the fiber axis 9
Cyclic CD/LD response on stretching along the fiber axis and sensor performance 10
4 scan method to isolate true CD 11
Electrospinning collector setup for spinning fibers 12
Chiroptical properties of PDMS films 13
Inhouse made tensile stage for studying chiroptical properties while stretching MSC/PDMS films 14
Changes in birefringence features on stretching PDMS and PDMS/MSC films........ 15
Calculations........ 16
Herman's orientation factor and orientation angle 16
Instrumental peak broadening in SAXS 16
Scherrer Calculation........ 17
Sensitivity of the strain sensor 18
4-scan CD measurement for isolating true CD 18
References........ 18

Figures and Tables

Viscosity and spinnability

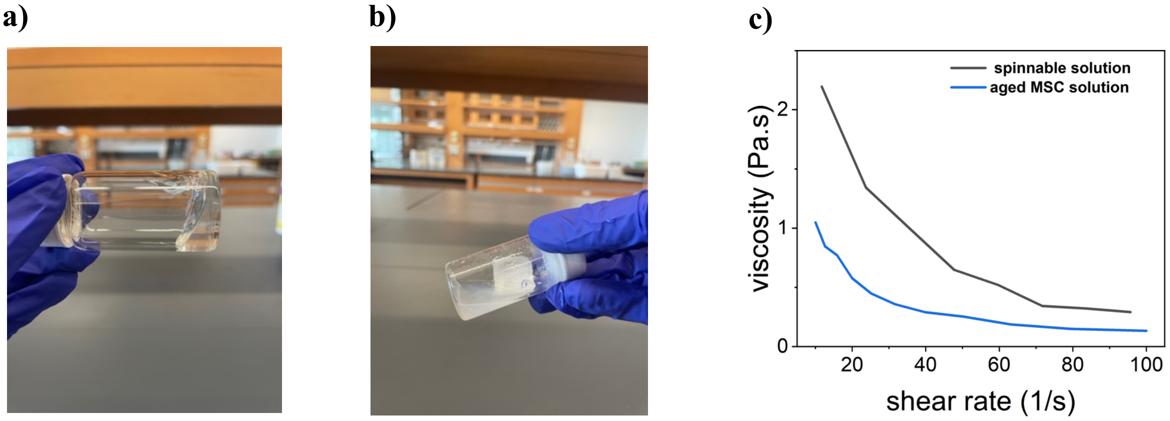

Figure S1. Photos showing the significant difference in viscosity of solutions from MSCs a) new batch (spinnable), b) aged batch (MSC dispersed in $CHCl_3$ after 3 months from synthesis and viscosity is low and results in spraying during electrospinning) and c) Viscosity vs shear rate measurements from rotational rheometric studies.

The carrier-free fiber formation is possible using CdS MSCs due to their lyotropic liquid crystalline polymer (LCP) behavior in moderately volatile non-polar solvents such as chloroform. The viscosity of MSC suspensions increases nonlinearly with increasing concentration and this effect alters the flow dynamics of fiber formation during the electrospinning process.[1] Both synthesis and storage conditions of MSCs have a significant effect on the rheological properties of suspensions and the ability to electrospin fibers. The viscoelastic data presented here are from suspensions prepared from equal concentrations of freshly synthesized, and aged MSCs (sealed in a desiccator for 3 months before preparing the suspension). Both suspensions show non-Newtonian (pseudoplastic) shear thinning behavior required for fiber formation, however, we were only able to achieve carrier-free electrospinning with freshly made samples due to its higher zero-shear viscosity.

Traditional electrospinning setup Vs. setup with rotary drum collector

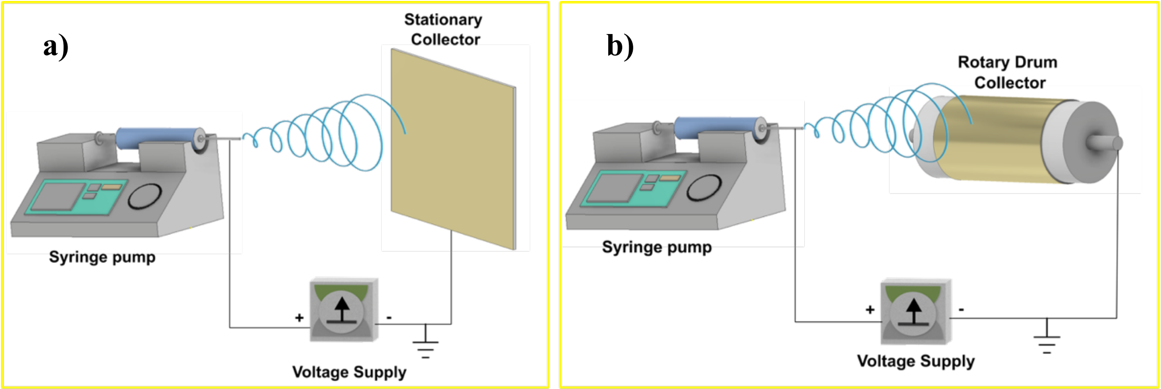

Figure S2. a)Traditional electrospinning setup, b) Electrospinning setup with rotary drum as the collector

Herman's orientation factor and orientation angle

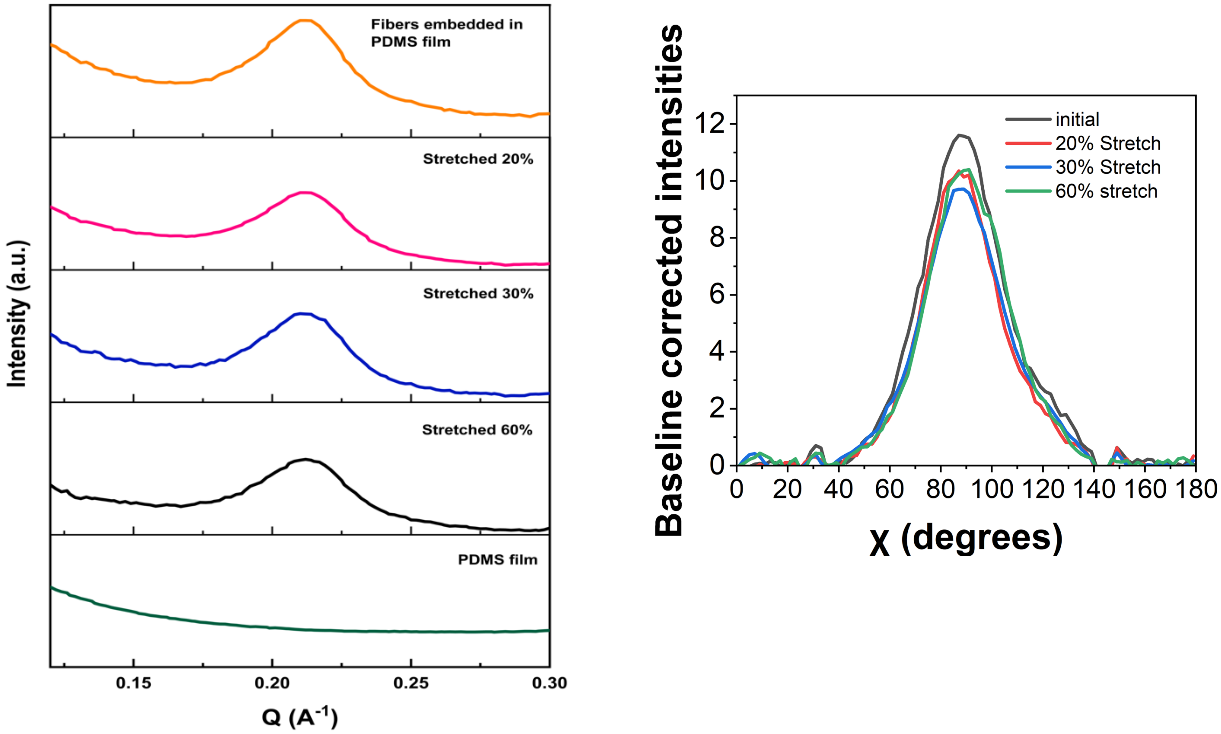

Figure S3. SAXS spectrum and the corresponding azimuthal scan spectrums of fibers embedded in PDMS and stretched at different levels.

Table S1. Herman's orientation factor and orientation angle for fibers embedded in PDMS - initial and stretched. The full width half maximum (FWHM) was measured using curve fitting function by using the software OriginPro 2023. The equation for the following is provided under Calculations section (equations S1-2).

**Sample**	**FWHM ($\Delta\varnothing_{1/2}$)**	**Orientation factor (f)**
Unstretched film with fibers	38.31$\pm$ 0.57	0.787$\pm$ 0.003
Stretched at 20%	35.98$\pm$ 0.58	0.800$\pm$ 0.003
Stretched at 30%	38.95 $\pm$ 0.53	0.784$\pm$ 0.003
Stretched at 60%	37.34$\pm$ 0.36	0.793$\pm$ 0.002

Ribbon shape of the electrospun fibers (SEM)

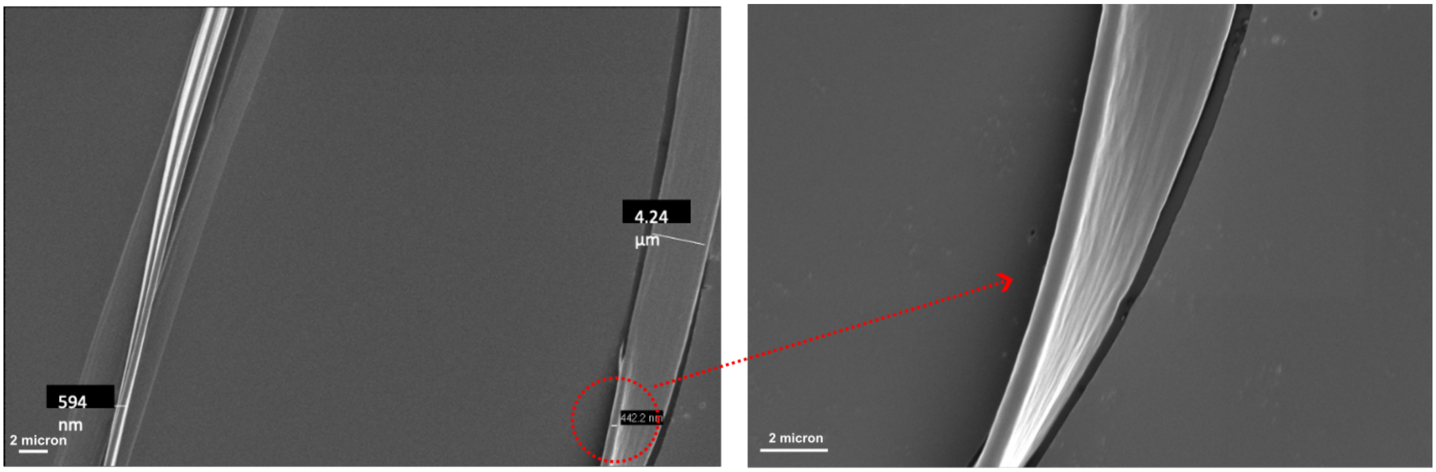

Figure S4. SEM images showing the flat ribbon like shape and the thick edges of the CdS MSC microfibers.

Isomers and Enantiomorphs of MSCs

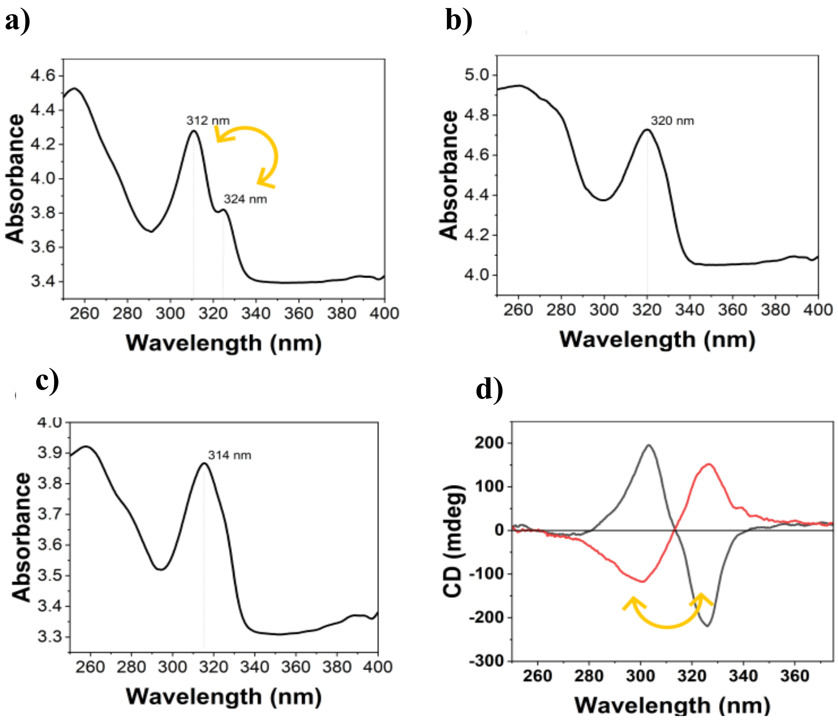

Figure S5. Two crystal forms are reported for CdS MSCs. Absorbance peaks suggesting the presence of a) both ß-CdS and α -CdS isomers, b) α -CdS form, c) ß-CdS form, d) CD spectrums for the two enantiomers reported for CdS MSCs (red- left handedness(+), black- right handedness (-))

The excitonic peaks at λ~313 nm and λ~324 nm is reported for ß-CdS and α -CdS isomers with zinc blende and wurtzite-like crystal phases, respectively.[2] Occasionally the CD transition (and absorption peak) is observed at λ ~322 nm. This absorption peak is slightly blue-shifted from λ ~324 nm observed for films,[3,4] but is likely due to effects of discontinuity and porosity of the fiber web. The excitonic peak at λ ~313 nm and λ ~324 nm is reported for ß-CdS and α -CdS isomers with zinc blende and wurtzite-like crystal phases, respectively.[2] The ß-CdS is said to be formed after the adsorption of water or other polar solvent on the surface of the cluster, due to hydrogen bonding with the oleate ligand and resulting structural reconfiguration and isomerization.[2] We confirm this transition takes place for electrospun samples due to the presence of the CD and UV-Visible absorbance peaks corresponding to ß-CdS and α-CdS isomers for fibers before and after conditioning (relative humidity 65% and temperature 21 °C) for 24 hours.

Chiroptical properties of fibers wound at 3200 rpm

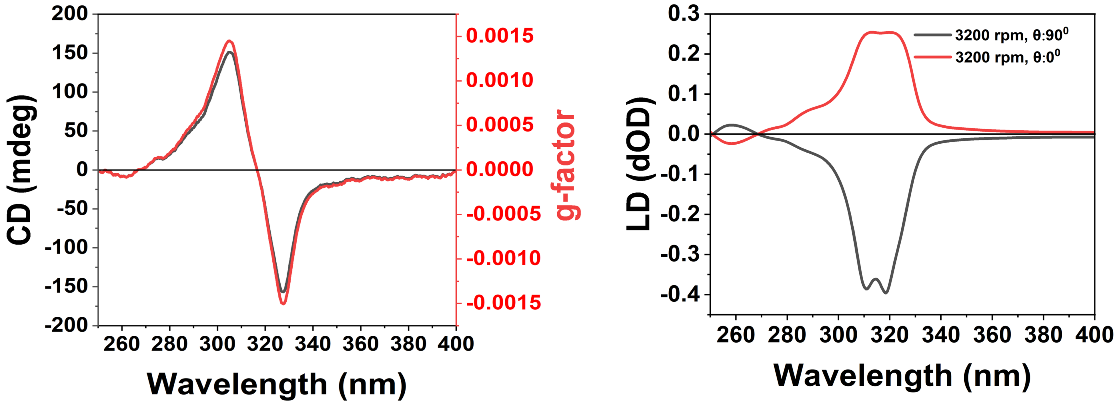

Figure S6. CD, LD and g-factor for electrospun MSC fibers using a winder rotating at 3200 rpm

The increase in LD (signifying the alignment of electronic transition dipole) matches with the macroscopic alignment of fibers seen in the FE-SEM images. The reason for the reduced macroscopic alignment (and relatively lower LD) for the highest rpm used (3,200 rpm) is likely due to fiber breakages at the higher rotational speed. Furthermore, the LD we observe for the randomly oriented fiber film, although less significant, is likely due to the alignment of mesophase along the fiber axis within the fibers.

Homochirality observed from a single batch of MSCs

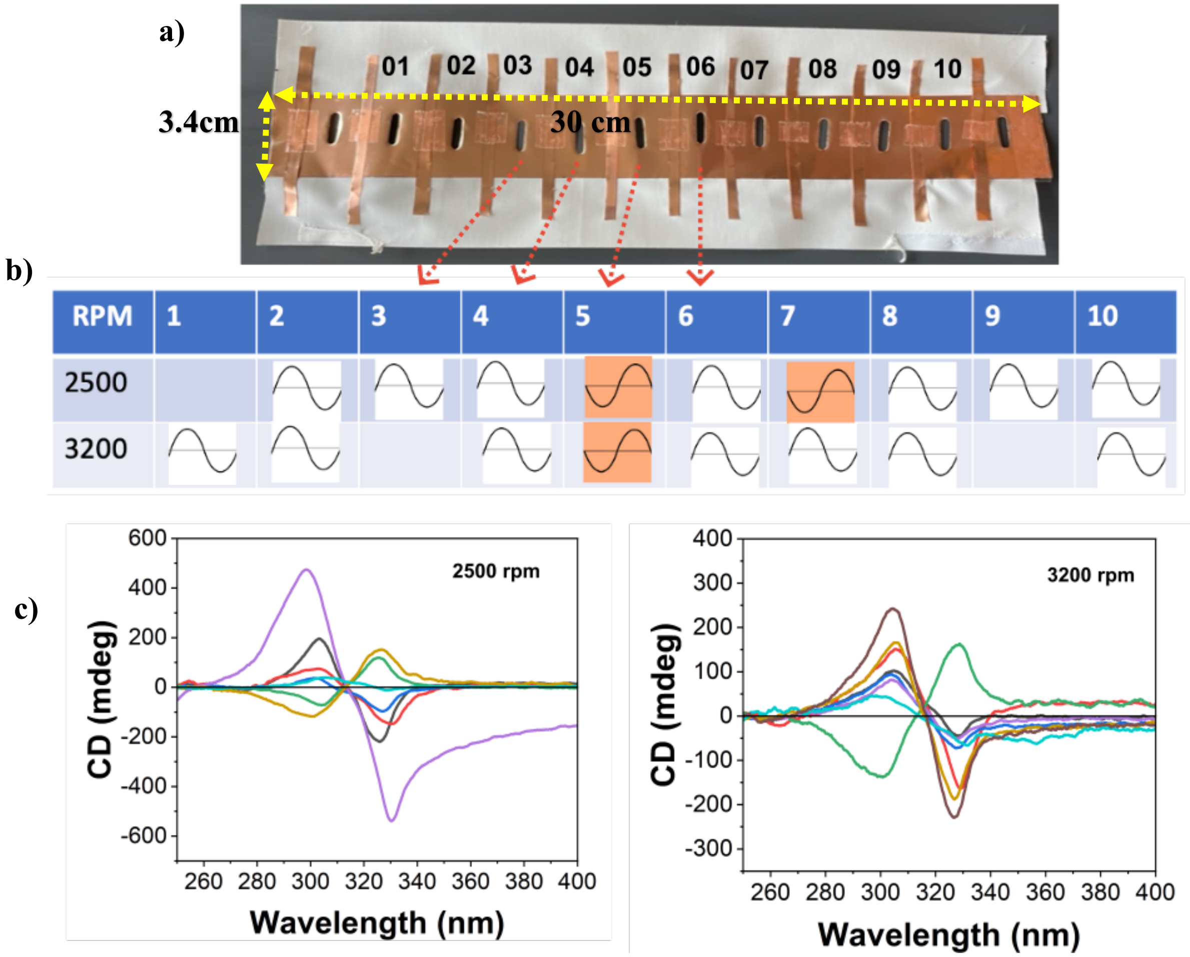

Figure S7. a) CD measurements were taken from multiple samples from one electrospinning run (approximately 40 min per run) . b-c) positive and negative CD bisignate shape and CD spectra of the fiber mats in each slit (for mats collected at 2500 and 3200 rpm)

The CD signal at each slit of the Copper sheet mostly resulted in the same handedness suggesting the presence of predominantly a single enantiomorph in one electrospinning cycle. The same handedness was observed even after the rpm of the collector was increased. However, the intensity of the CD signal is different due to the variations in thickness of the fiber mat at different slits.

Chiroptical response on stretching the MSC/PDMS film across the fiber axis

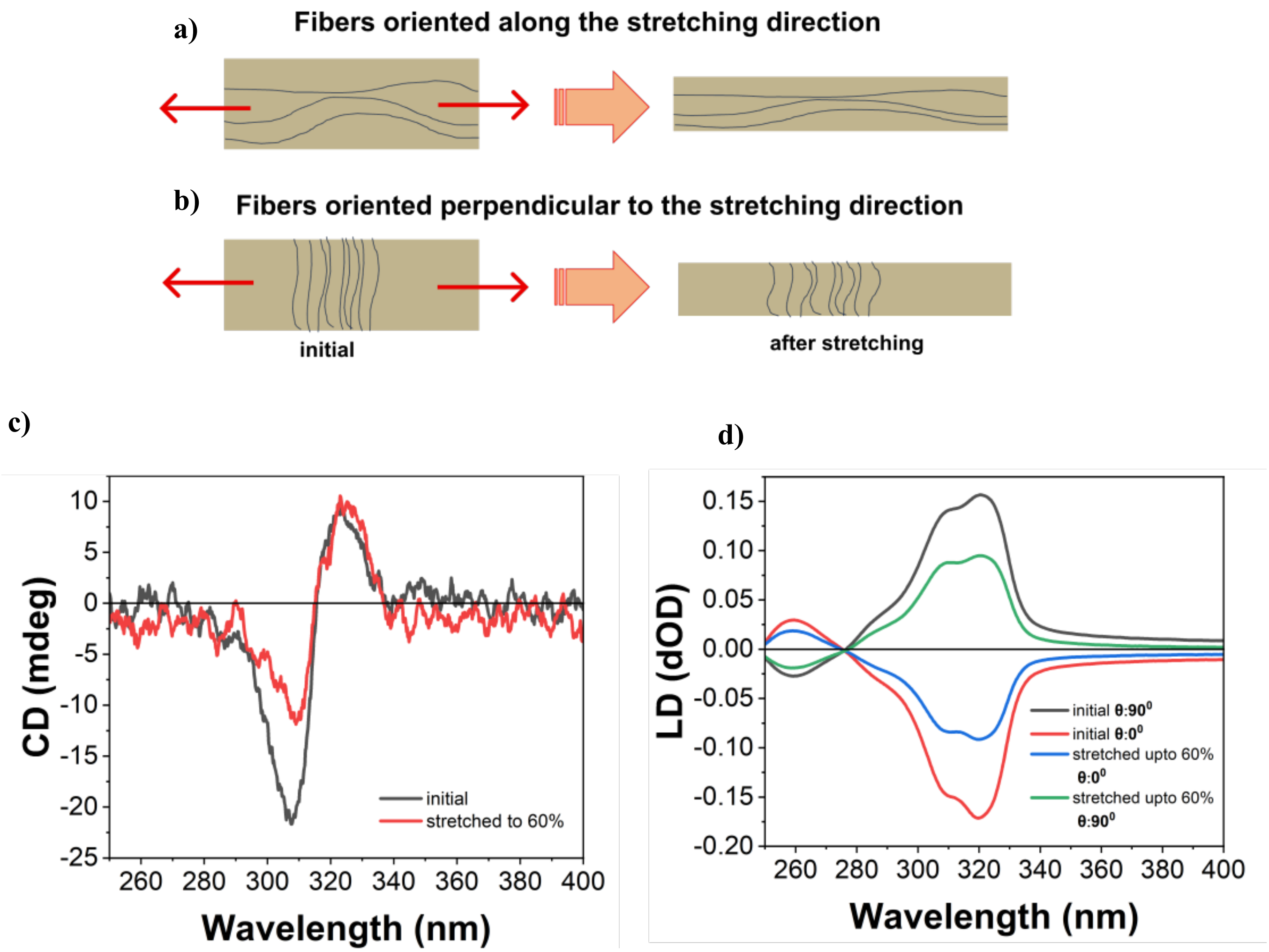

Figure S8. Schematic of stretching of the PDMS films with fibers a) along and, b)across the fiber axis, c-d) CD and LD of fibers embedded in PDMS film – initial vs stretching up to 60% with the fiber axis perpendicular to stretching direction. All experiments mentioned in the main text involves stretching along the fiber axis.

Cyclic CD/LD response on stretching along the fiber axis and sensor performance

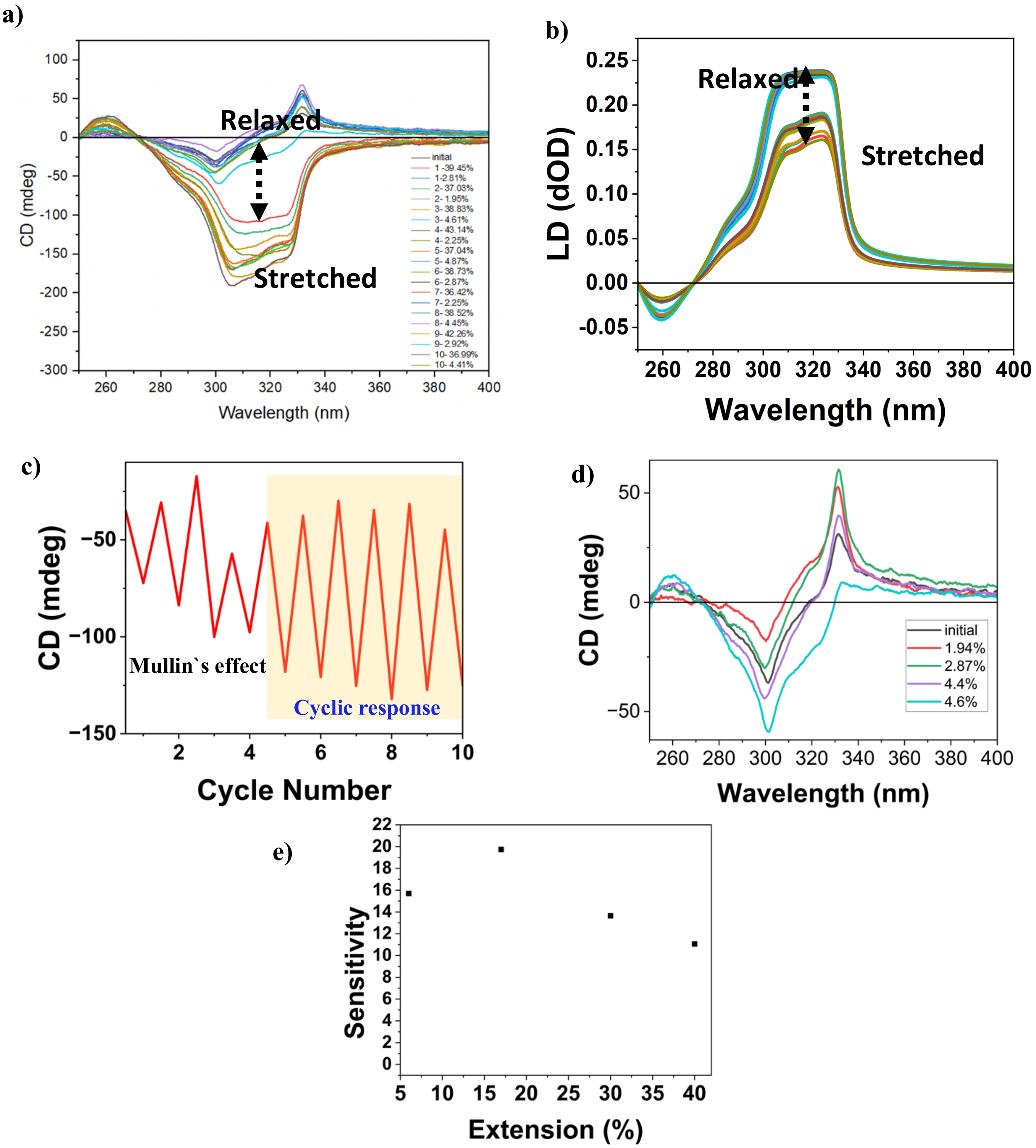

Figure S9. CD and LD for fibers embedded in PDMS film (length 2 cm) repeatedly stretched to 40±2% and relaxed to original length for 10 cycles, c) change in CD magnitude at λ ~ 310 nm over the ten cycles. Over the first 4 cycles the film undergoes relaxation (Mullin`s effect), d) detection limit and ambiguity at lower extensions <4.5%, e) Sensitivity of the sensor plotted against the strain (equation S5 provided under calculations).

4 scan method to isolate true CD

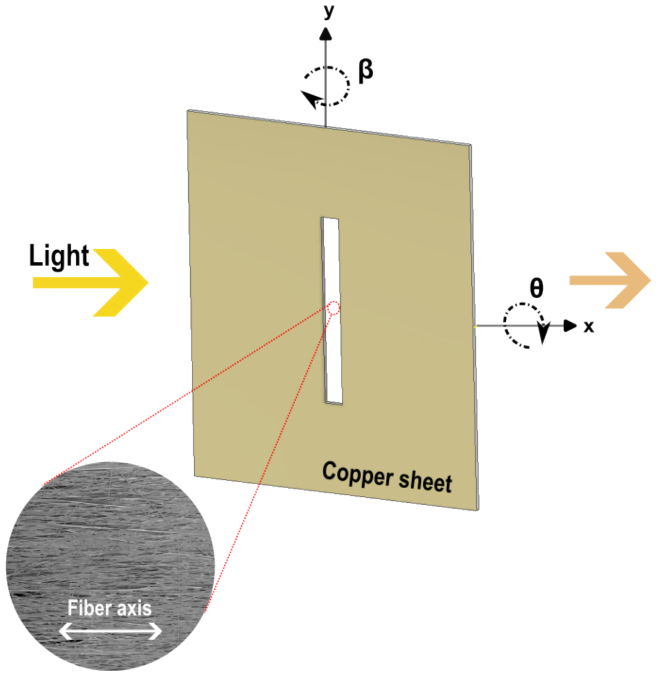

Figure S10. Schematic showing the inversion and rotation of the fiber mat electrospun on copper sheet with a slit to take CD and LD measurements for the 4 scan method. Magnified SEM image shows the uniaxial orientation of the fibers along the x axis when θ = 0° (electrospun on drum collector rotating at 2500 rpm)

The 4 scan method makes use of the antisymmetric properties to remove the contribution of linear anisotropies to the CD signal by measuring four different orientations, (1)θ=0°, β=0° (2)θ=90°, β=0° (3)θ=0°, β=180° and (4)θ=90°, β=180° and averaging over the four scans. Changes in θ refer to whether fibers are aligned horizontally or vertically, while β refers to whether fibers are facing towards or away from the UV source of the CD spectrometer. These orientations are graphically represented in above Figure S10. The equations S6-9 in the calculations section are used to plot the true CD, CD_{α} (CD generated by PEM), and CD $_{LDLB}$ considering 4 scan CD data of the films before and after stretching (Figure 3).

Electrospinning collector setup for spinning fibers

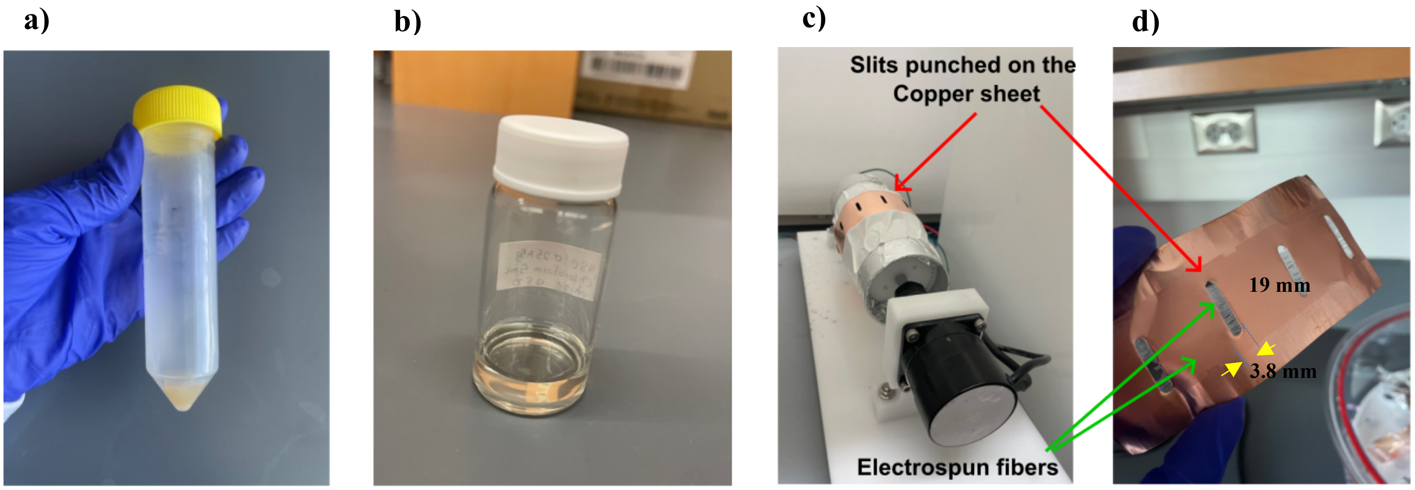

Figure S11. a) Solid MSC sample, b)MSC dispersed in $CHCl_3$, c) Copper sheet with slits wrapped around drum collector of the electrospinning setup, d) aligned fibers formed in the slits and on the copper sheet

Chiroptical properties of PDMS films

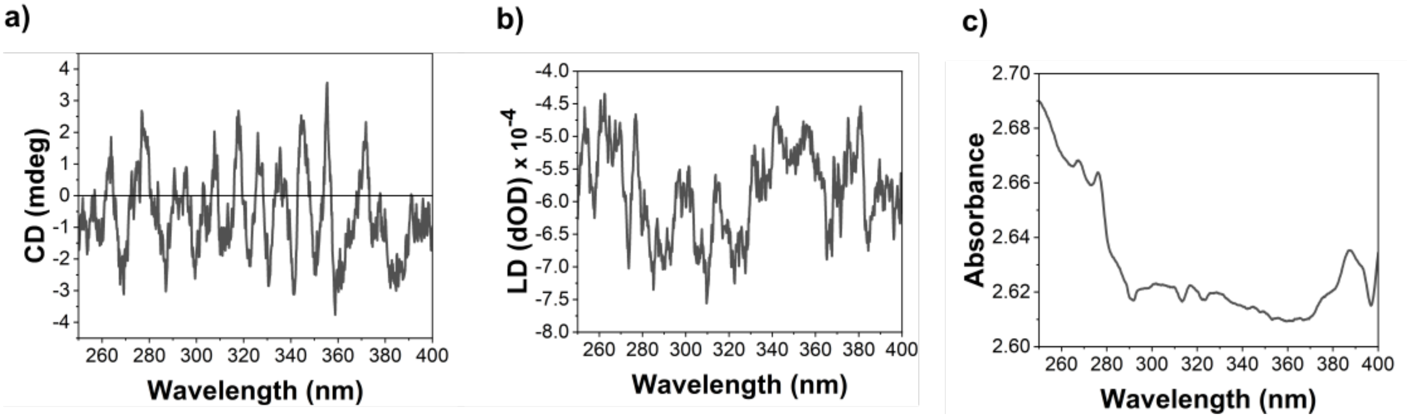

Figure S12. a) CD, b) LD, and c) absorbance spectra of a thin PDMS film (0.7 mm)

The inherent mechanical properties of the electrospun fiber mat does not allow it to behave as a standalone device. Therefore, we imbibe the aligned fibers in a polydimethylsiloxane (PDMS) based film since the polymer itself is achiral, does not add CD or LD contributions, and shows no photoelastic properties nor absorption in the UV region. Literature also mentions chirality amplifications when a PDMS film was superimposed with another chiral film due to the photo reflectivity of PDMS.[5]

Inhouse made tensile stage for studying chiroptical properties while stretching MSC/PDMS films

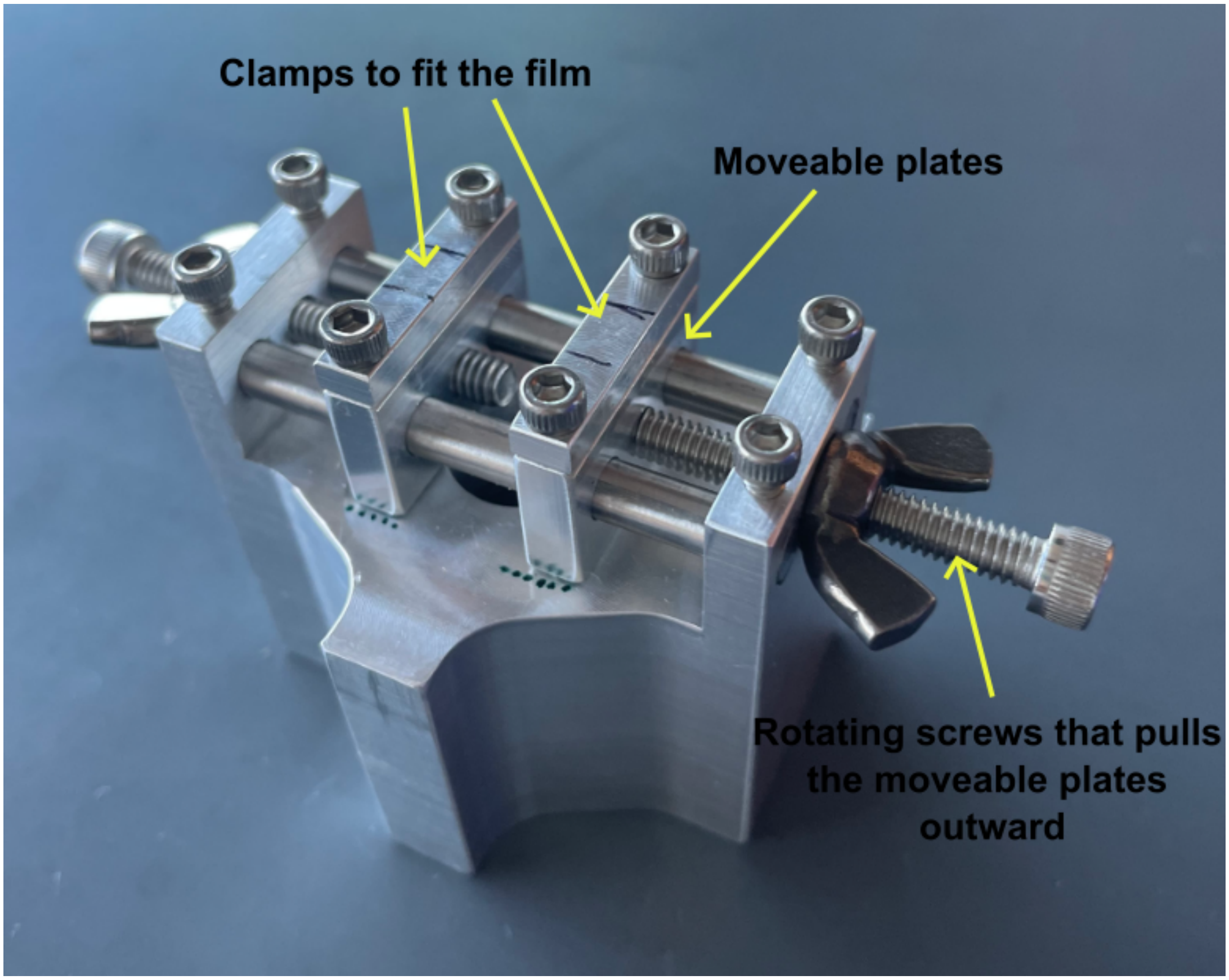

Figure S13. A tensile stage that can fit onto the sample stage of the CD spectrometer in order to take measurements of stretched PDMS films with MSC fibers.

Changes in birefringence features on stretching PDMS and PDMS/MSC films

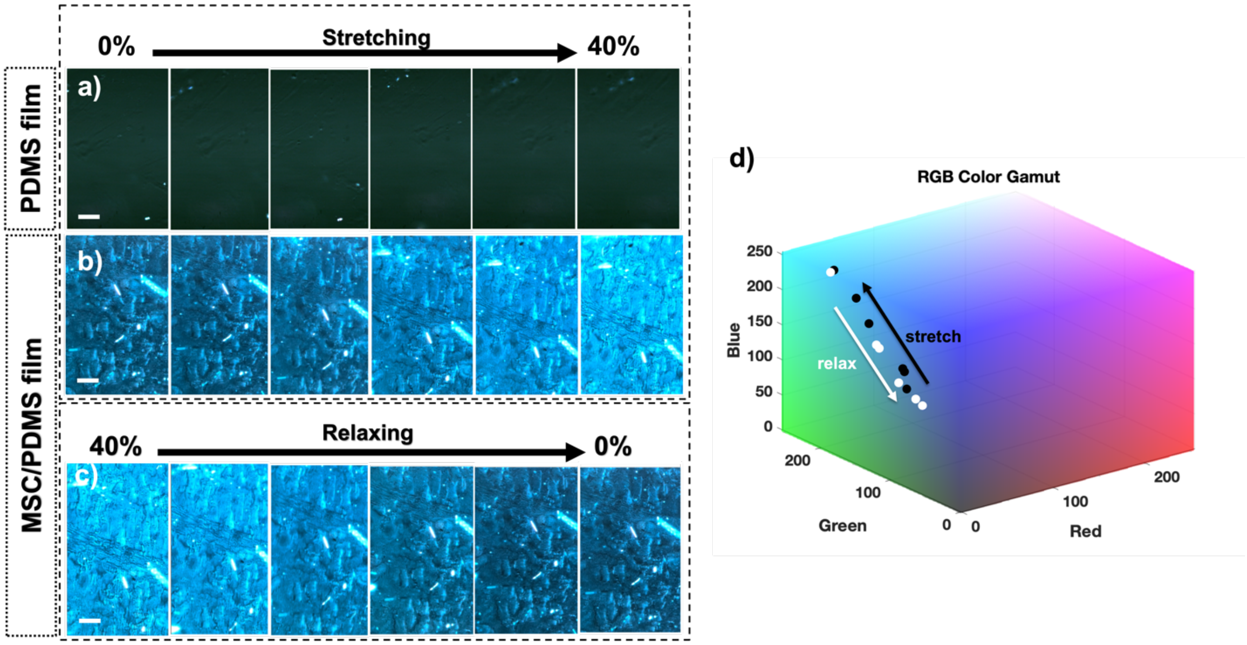

Figure S14. a) changes in birefringence features on stretching the PDMS film, b-c) changes in birefringence features on reversible stretching of the MSC/PDMS film d) RGB coordinates of the color change observed while PDMS/MSC films are stretched (black circles) and relaxed (blue circles) are plotted on the RGB color gamut.

Calculations

Herman's orientation factor and orientation angle

The following equations were used to quantify the orientation of the mesophase along the fiber axis. 'f' is referred as the Herman's orientation factor. Compared to the more robust method generally used, please note that the following equation S1, is a convenient approximation [6]. We have however, validated the calculated values using the conventional method given by equation S2 below.

$$\mathrm{f} = \frac{180^0 - \Delta\emptyset_{1/2}}{180^0} \qquad \textbf{(S1)}$$

Where $\Delta\emptyset_{1/2}$ is the FWHM of the azimuthal scan in degrees.

$$\mathrm{f} = \frac{3\langle\cos^2\emptyset\rangle - 1}{2} \qquad \textbf{(S2)}$$

Where;

$$\langle\cos^2\emptyset\rangle = \frac{\int_{-\frac{\pi}{2}}^{\frac{\pi}{2}} \mathrm{I}(\emptyset)\cos^2\emptyset\sin\emptyset\, d\emptyset}{\int_{-\frac{\pi}{2}}^{\frac{\pi}{2}} \mathrm{I}(\emptyset)\sin\emptyset\, d\emptyset}$$

Instrumental peak broadening in SAXS

The model mentioned by Zhang et al.[7] was used to calculate the peak broadening. First, the scattering from the silver behenate standard is fitted to get the peak positions and widths of the first three orders of reflections. The fitting was done with an exponential background and three Gaussian peaks (Figure S15a). The Gaussian widths were converted to FWHM by multiplying by 2.355. Then the peak FWMH versus the position was plotted and extrapolated to q=0 $Å^{-1}$ to estimate the instrument broadening (Figure S15b). The Δq_{res} was approximated to be 0.0042 $Å^{-1}$.

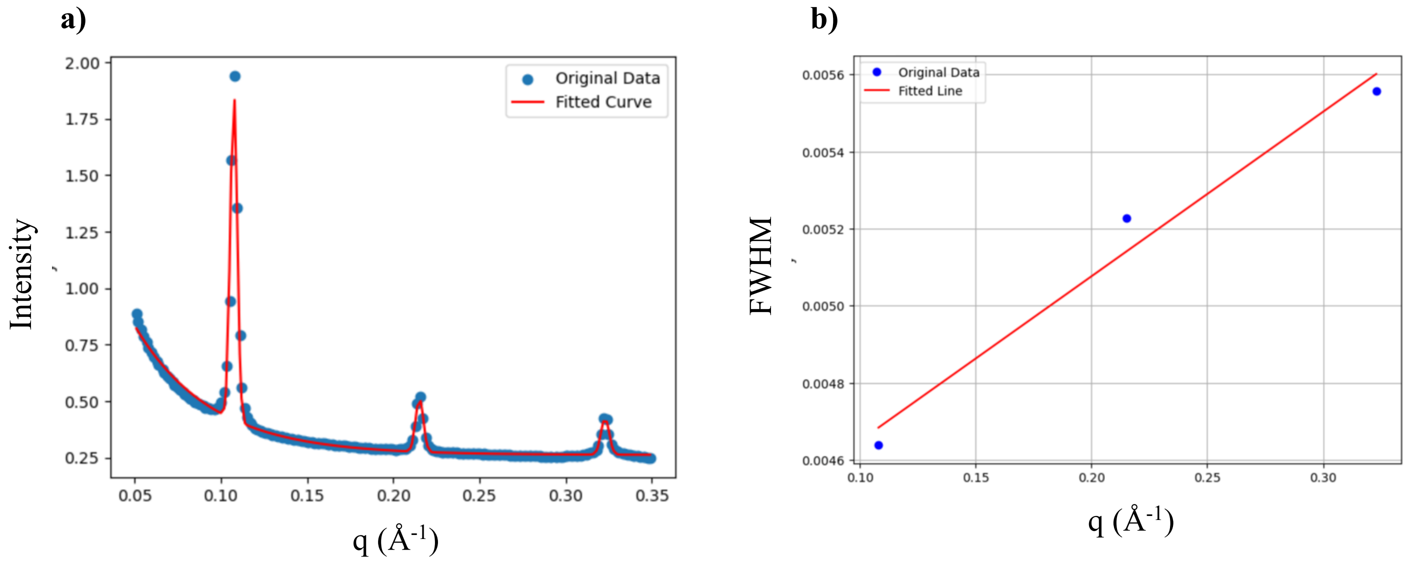

Figure S15. a) Gaussian fitting of the peaks, b) the peak FWHM vs the q position

The total measured peak, Δq_{meas} for the unstretched sample is determined to be approximately 0.04 $Å^{-1}$ by fitting the scattering. The following equation S3 is used to determine the broadening due to the scatterers in the sample, Δq_{sample}. The peak broadening is approximated to be 11%.

$$\Delta q_{sample} = \sqrt{(\Delta q_{meas})^2 - (\Delta q_{res})^2} \quad \textbf{(S3)}$$

$$\Delta q_{sample} = \sqrt{(0.04)^2 - (0.0042)^2} \quad = 0.039 \text{ Å}^{-1}$$

Scherrer Calculation

The q-form of the Debye-Scherrer equation is used to calculate the grain size.

$$\xi = \frac{2\pi K}{\Delta q_{sample}} \quad \textbf{(S4)}$$

where K is a shape factor constant and ξ is the grain size. Couple of values of K (1, 0.939, and 0.9) was used to determine the grain sizes. The resulting grain sizes were 160 Å, 150 Å, and 144

Å, respectively. Therefore, conservatively, the grain size (or correlation length) of the mesophases is on the order of 150 Å or 15 nm.

Sensitivity of the strain sensor

$$Sensitivity = \frac{\frac{CD - CD_0}{CD_0}}{\varepsilon} \quad \textbf{(S5)}$$

Where CD and ε refers to the CD and extension, respectively with the subscript 0 referring to initial measures.

4-scan CD measurement for isolating true CD

$$CD_{measured} = CD_{true} + CD_{LDLB} + CD_{\alpha} \quad \textbf{(S6)}$$

$$CD_{true} = CD_{four\ scan\ average} =[CD_{\theta=0°,\ \beta=0°} + CD_{\theta=0°,\ \beta=180°} + CD_{\theta=90°,\ \beta=0°} + CD_{\theta=90°,\ \beta=180°}]/4 \quad \textbf{(S7)}$$

$$CD_{\alpha,\ \theta=0°}= [CD_{\theta=0°,\ \beta=0°} + CD_{\theta=0°,\ \beta=180°} - 2CD_{four\ scan\ average}]/2 \quad \textbf{(S8)}$$

$$CD_{LDLB,\ \beta=180°} = [CD_{\theta=0°,\ \beta=180°} + CD_{\theta=90°,\ \beta=180°} - 2CD_{four\ scan\ average}]/2 \quad \textbf{(S9)}$$

References

[1] H. Han, K. Hirsch, T. Hanrath, R. D. Robinson, L. M. Shepherd, *Adv Eng Mater* **2021**, *23*, 1.

[2] C. B. Williamson, D. R. Nevers, A. Nelson, I. Hadar, U. Banin, T. Hanrath, R. D. Robinson, *Science (1979)* **2019**, *363*, 731.

[3] D. R. Nevers, C. B. Williamson, B. H. Savitzky, I. Hadar, U. Banin, L. F. Kourkoutis, T. Hanrath, R. D. Robinson, *J Am Chem Soc* **2018**, *140*, 3652.

[4] H. Han, S. Kallakuri, Y. Yao, C. B. Williamson, D. R. Nevers, B. H. Savitzky, R. S. Skye, M. Xu, O. Voznyy, J. Dshemuchadse, L. F. Kourkoutis, S. J. Weinstein, T. Hanrath, R. D. Robinson, *Nat Mater* **2022**, *21*, 518.

[5] L. Song, K. Yang, B. Zhao, Y. Wu, J. Deng, *ACS Appl Mater Interfaces* **2023**, *15*, 4601.

[6] T. Kongkhlang, K. Tashiro, M. Kotaki, S. Chirachanchai, *J Am Chem Soc* **2008**, *130*, 15460.
[7] R. Bin Zhang, G. Ungar, X. Zeng, Z. Shen, *Soft Matter* **2017**, *13*, 4122.
